# Cataract surgery at Aravind Eye Hospitals: 1988–2008

**Published:** 2008-09

**Authors:** G Natchiar, RD Thulasiraj, R Meenakshi Sundaram

**Affiliations:** Vice Chair & Director – Human Resources, Aravind Eye Care System, Anna Nagar, Madurai 625 020, Tamil Nadu, India.; Executive Director, Lions Aravind Institute of Community Ophthalmology, Aravind Eye Care System; Past President, VISION 2020: The Right to Sight: India.; Senior Manager – Outreach, Aravind Eye Care System.

**Figure:**
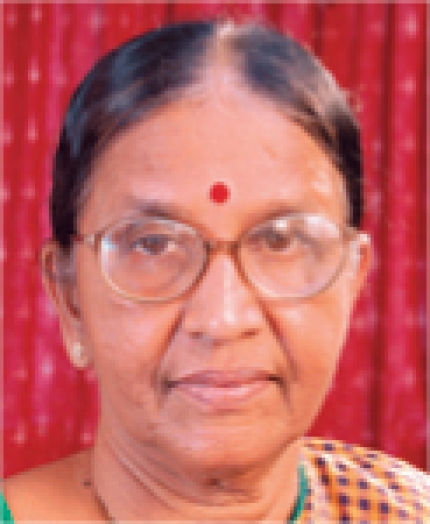


**Figure:**
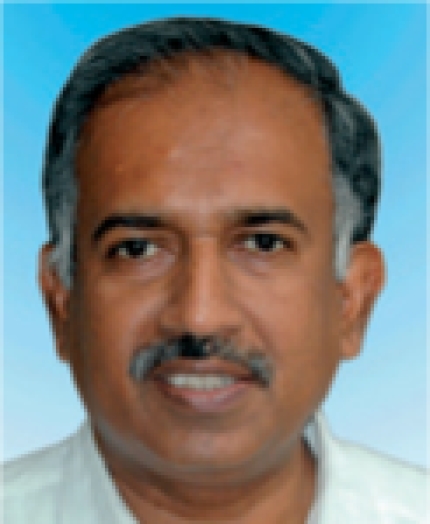


**Figure:**
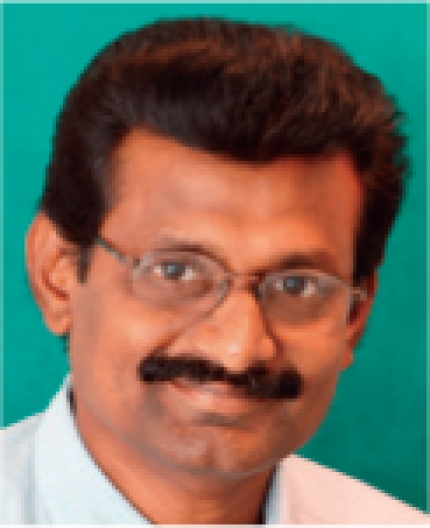


In the 1980s, cataract was the major cause of blindness in India and was responsible for 80% of all blindness.^1^^,^^2^ This prompted the Indian government to launch a national cataract control programme, which succeeded in lowering the prevalence of blindness from 1.49% to 1.1%.^2^^,^^3^ In addition, by 2000, this programme had reduced the proportion of people blind due to cataract from 80% to 62%.^4^

Aravind Eye Hospitals contributed to a third of all cataract operations in the state of Tamil Nadu during the last two decades and played a major part in lowering the rate of blindness in that state. By 2000, the prevalence level of blindness was just 0.78%, compared to the national level of 1.11%.^3^

The first Aravind Eye Hospital was founded in 1976 and contained just 11 beds. There are now five Aravind Eye Hospitals, located up to 500 km apart, which form part of Aravind Eye Care System (AECS). Over the last two decades, the organisation has increased six-fold the number of cataract operations it performs annually: from a total of 29,928 in 1988 to 180,991 operations in 2007, performed at the five centres.

**Figure:**
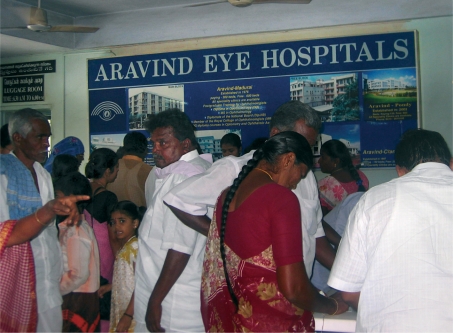
The Aravind Eye Hospital in Madurai. INDIA

During the last two decades, over 70% of the cataract operations on poor patients have been performed free of charge or at a heavily subsidised rate; the other 30% have been conducted in the separate paying section, on patients who could afford the regular fee.

## Transition in cataract services: 1988–2008

### Community outreach

From the day the hospital opened its doors in 1976, Aravind has been organising community outreach, always with the involvement of the local community. This partnership has led to widespread awareness of cataract services across the state of Tamil Nadu. Today, the same strategy is being used successfully to create awareness about other conditions, such as diabetic retinopathy, refractive error, and childhood blindness, and to address them.

In the late 1970s, surgical eye camps were in vogue in India. During this era, in addition to the hundreds of screening camps it held, Aravind Eye Hospital organised a few surgical eye camps, which proved to be very resource intensive. In these surgical camps, operations were performed at the site, which could be a school, a college, a community hall, or a local hospital. At that time, intracapsular cataract extraction (ICCE) was the chosen surgical procedure. The postoperative stay at the camp site ranged from four to seven days. Patients had to lie down with their eyes bandaged in a complete resting position to avoid wound leak or iris prolapse, and they were given soft food to eat. The operated patients were issued standard +10 D aphakic spectacles at the time of discharge and were advised to come for follow-up at the base hospital or camp site a month later.

We reduced the number of surgical eye camps over the years and, in 2002, we completely stopped organising them, as the growing network of Aravind Eye Hospitals provided easy access for the community in each service area. Screening eye camps, however, continue to this day; the patients brought in from these camps account for over 50% of the cataract operations performed in the five Aravind Eye Hospitals.

**Figure:**
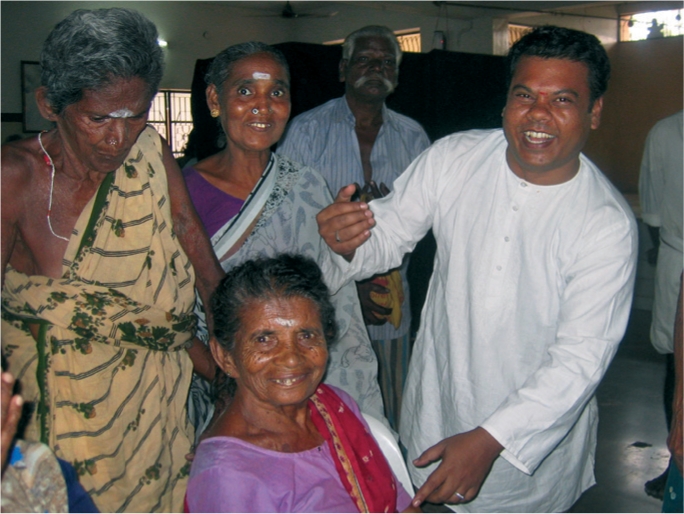
Older patients are welcomed at a screening camp. INDIA

### Indications for cataract surgery

In 1988, the selection criteria for cataract surgery was best corrected visual acuity (VA) of <6/60. In the 1990s, as new surgical techniques started to result in better visual rehabilitation, the criteria changed to include patients who, due to cataract, found it difficult to perform their daily tasks. This marked a shift away from clinical criteria and towards more patient-centred criteria. In addition, more and more patients are spontaneously coming for early surgery before they become blind, since cataract operations now have very good outcomes.

### Quality assurance

In the 1980s, since only patients with a preoperative vision ranging from light perception to VA <6/60 were selected, patients were very happy with their postoperative vision, in spite of the limitations of the aphakic spectacles they were given. Nowadays, as patients with much better preoperative vision are being admitted, it has become essential to assess the quality of postoperative vision as well. In addition to quantitative visual acuity assessment, patients are also asked how satisfied they are with their vision. Amongst other things, this helps to refine postoperative refractive error correction by getting a better sense of the patients' expectations; it also helps in explaining to patients about the vision recovery process and what to expect. This improved vision assessment is a reflection of the modern lifestyle in India, which demands a much better quality of vision.^5^

At Aravind, surgical complications and the outcomes of every operation have been monitored since 1991, using continually evolving software, to enhance quality by analysing visual outcomes, infections, complications, and the number of patients needing a second operation on the same eye. This has helped put in place a system of continuous improvement.^6^ For example, out of 73,323 cataract operations performed in 2007 at the Aravind Eye Hospital in Madurai, the rate of surgical complication was 1.6%, the rate of postoperative infection was 0.05%, and corrective surgery was needed on the operated eye in 0.4% of cases.

## High-volume cataract surgery

Aravind's success at performing a large number of cataract operations per year and per surgeon (known as high-volume cataract surgery) is based on three main pillars:

- making intraocular lenses more affordable
- training cataract surgeons
- developing good systems of service delivery (as described above), as well as innovative operating practices (the ‘assembly line’ system, described below).

### Making intraocular lenses more affordable

Although ICCE was the standard procedure at Aravind at the time, some surgeons had started to use ‘iris clip’ intraocular lenses (IOLs) in the paying section as early as 1979; a total of 20 such lenses were used in that year. A switch to anterior chamber IOLs was made in 1981 and, by the mid-1980s, surgeons started to use posterior chamber lenses regularly.

IOLs were however very expensive (imported at US $100 each in the mid-1980s) and these operations could only be offered in the paying section. The hospital therefore could not afford to give them away free or at low cost to poorer patients. For the same financial reasons, international nongovernmental development organisations (NGDOs), as well as the Indian government, did not support IOL surgery at the time.

However, Dr G Venkataswamy, the founder of Aravind Eye Hospitals and AECS, felt strongly that every villager undergoing cataract surgery should get an IOL implant. He understood how difficult it was for these people to carry out farm work with aphakic spectacles. The major obstacles to offering IOL implantation to everyone were the high cost of IOLs and the fact that there were not enough ophthalmologists trained to perform this type of surgery. In addition, traditional operating practices needed to be assessed and improved, if necessary.

**Figure:**
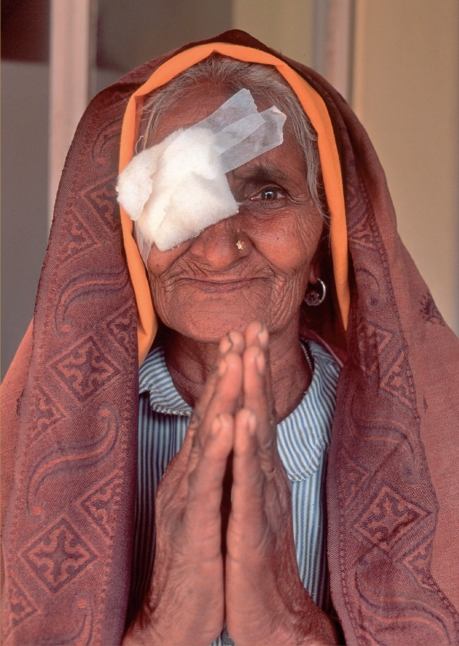
The decision, in 1992, to locally produce high-quality IOLs and the introduction of manual SICS techniques in 1998 have contributed to much greater satisfaction amongst patients, both paying and non-paying

In 1992, AECS established Aurolab, a local non-profit trust, with the support of the Seva Foundation, Combat Blindness Foundation, and Sightsavers International. Aurolab undertook the task of producing low-cost IOLs. These were initially sold for US $10 each. The price of the IOLs produced by Aurolab steadily declined as efficiency and demand increased; at present, the cost of rigid polymethyl methacrylate (PMMA) IOLs stands at around US $2. These have been made available in over 120 developing countries. The trust also started to produce sutures, pharmaceuticals, and other surgical supplies locally. This has helped to bring down the cost of cataract operations further in India and elsewhere.

In 1994, Aravind introduced phacoemulsification. Considering the very good outcome of this new, sutureless IOL technique, Aravind promoted it very intensively amongst the paying patients who could afford the additional cost.

The development of the manual, sutureless, small incision cataract surgery (SICS) represented another great stride towards offering IOL cataract surgery to more patients. SICS is cheaper, quicker, and easier to perform, and its outcomes compare very well with that of phacoemulsification. In 1998–1999, we introduced this technique at Aravind in both the ‘free or subsidised’ and paying sections (see Table 1).

**Table 1:**
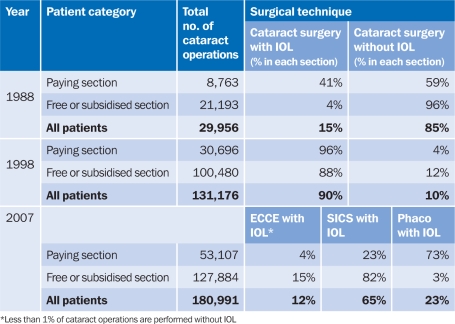
Surgical techniques used for cataract operations over the last two decades at Aravind Hospitals

### Training cataract surgeons

To address the challenge of developing the skills required for microsurgery with IOL implants, AECS designed and started the Micro Surgery Training Programme in 1993, with support from Sightsavers International.

To promote high-quality and high-volume cataract surgery, Aravind also published a series of manuals, such as the *Quality Cataract Series* in 2001, the *Manual on SICS technique* in 2000, and the *Manual for IOL trainees* in 2001.

As of August 2008, a total of 1,622 trainees from 44 countries have been trained at Aravind: 1,132 in ECCE, 310 in SICS, and 180 in phacoemulsification (see Table 2).

**Table 2:** Worldwide distribution of Aravind trainees by World Health Organization Regions

	Europe	Americas	South East Asia	Africa	Western Pacific	Eastern Mediterranean
Candidates trained at Aravind in IOL surgery	91	10	1,353	38	101	29

### Developing innovative operating practices

In order to ensure a high volume of cataract operations, while keeping the quality of surgery high, it is vital to use the time of the ophthalmologist as effectively as possible. Indeed, ophthalmologists are probably the most expensive and scarce resource needed to perform a cataract operation.

Aravind developed specific practices to increase the volume of cataract operations. These are known as the ‘assembly line’ system and consist of the following three elements:

- setting up an efficient patient flow
- organising the operating equipment and support staff to match the output capacity of the surgeons
- ensuring that all surgical supplies are available and that equipment is in good working condition.

**Figure:**
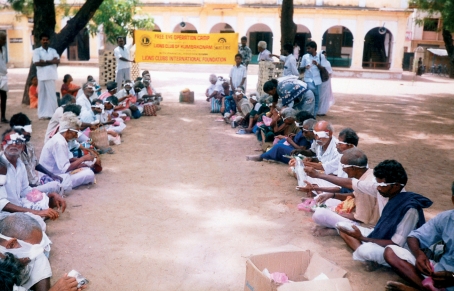
Patients discharged from a surgical camp are given food before they return home. INDIA

Each day of surgery is planned meticulously in advance. This includes planning the number of surgeons and nurses needed, as well as the number of IOLs and other surgical supplies required. On the day of his/her operation, the patient receives the prescribed local anaesthesia and is then led to the operating room, where each surgeon has two operating tables. The patient is draped and prepared on one table, while the surgeon is operating on the other table, on a patient admitted earlier. On completing the operation, the surgeon swings the arm of the microscope over the next table, follows the prescribed asepsis protocol, and then begins surgery on the new patient. Table 3 illustrates how the productivity of a single surgeon increases according to the availability of operating equipment and support staff.

**Table 3:** The impact of operating equipment and support staff on the number of cataract operations a single surgeon can perform in one hour at Aravind Eye Hospitals

Operating tables	Scrub nurses	Running nurses	instrument sets	Operations per hour
1	1	1	1	1
1	1	1	2	2
2	2	1	6	6–8

## Conclusion: the impact of the Aravind model

The developments described in this article have all played a major role in increasing the cataract surgery rate in Tamil Nadu (from 2,039 in 1988–9 to 7,633 in 2005–06), and in India as a whole.

The production of cheaper intraocular lenses by AECS and the establishment of a training programme for ophthalmologists have also increased the number of high- quality cataract operations performed worldwide. This is particularly true in developing countries, where costs were prohibitive before the introduction of low-cost IOLs.

Aravind has shown that cataract operations can be done on a massive scale, while still providing quality care. Previously, there was one kind of surgery for the upper classes and another for the masses. History has shown that, with appropriate technology and processes (the assembly line system, locally made IOLs and consumables, and locally trained surgeons), it is possible to duplicate developed world results at an affordable cost.

**Current practice at Aravind**

**In the paying section:**

- Around 75% of cataract operations are performed using phacoemulsification, mostly with foldable lenses. Many of the patients go home within two hours of surgery. A total of 80% of operations are performed under topical anaesthesia.
- Around 25% of operations are done using manual, sutureless small incision cataract surgery (SICS) techniques (usually when the nucleus is very hard).
- Less than 5% of operations are done using extracapsular cataract extraction (ECCE) when phacoemulsification or SICS are contraindicated.

**In the ‘free or subsidised’ section**:

- 80% of walk-in patients and those referred from eye camps are operated using manual, sutureless SICS techniques. Patients go home either on the same day or the next day, depending on the distance they need to travel. These operations are done under peribulbar or retrobulbar anaesthesia. This has increased the productivity of surgeons and brought down the cost of surgery.
- In cases where SICS is not possible, ECCE with IOL is performed.
- Almost 99% of cataract operations are performed with IOL.
